# High tacrolimus blood concentrations early after lung transplantation and the risk of kidney injury

**DOI:** 10.1007/s00228-017-2204-8

**Published:** 2017-01-28

**Authors:** M. A. Sikma, C. C. Hunault, E. A. van de Graaf, M. C. Verhaar, J. Kesecioglu, D. W. de Lange, J. Meulenbelt

**Affiliations:** 1 Department of Intensive Care Medicine, University Medical Center Utrecht, Utrecht, The Netherlands; 2 Dutch Poisons Information Center, University Medical Center Utrecht, Utrecht, The Netherlands; 3 Department of Intensive Care Medicine and Dutch Poisons Information Center, Division of Anesthesiology, Intensive Care and Emergency Medicine, University Medical Center Utrecht, F06.149, P.O. Box 85500, 3508 GA Utrecht, The Netherlands; 4 Department of Lung Transplantation, University Medical Center Utrecht, Utrecht, The Netherlands; 5 Department of Nephrology and Hypertension, University Medical Center Utrecht, Utrecht, the Netherlands; 6 Institute for Risk Assessment Sciences, University Medical Center Utrecht, Utrecht, The Netherlands

**Keywords:** “tacrolimus”[MeSH Terms] OR tacrolimus[Text Word], “lung transplantation” [MeSH Terms] OR lung transplantation[Text Word], Nephrotoxic[All Fields] OR nephrotoxicant[All Fields] OR nephrotoxicity[All Fields] OR nephrotoxin[All Fields], “acute kidney injury”[MeSH Terms] OR acute kidney injury[Text Word], “pharmacokinetics”[Subheading] OR “pharmacokinetics”[MeSH Terms] OR pharmacokinetics[Text Word]

## Abstract

**Purpose:**

Lung transplant recipients often develop acute kidney injury (AKI) evolving into chronic kidney disease (CKD). The immunosuppressant tacrolimus might be associated with the emergence of AKI. We analyzed the development and recovery of kidney injury after lung transplantation and related AKI to whole-blood tacrolimus trough concentrations and other factors causing kidney injury.

**Methods:**

We retrospectively studied kidney injury in 186 lung-transplantation patients at the UMC Utrecht between 2001 and 2011. Kidney function and whole-blood tacrolimus trough concentrations were determined from day 1 to 14 and at 1, 3, 6, and 12 months postoperative. Systemic inflammatory response syndrome (SIRS), septic shock, and nephrotoxic medications were evaluated as covariates for AKI. We analyzed liver injury and drug-drug interactions.

**Results:**

AKI was present in 85 (46%) patients. Tacrolimus concentrations were supra-therapeutic in 135 of 186 patients (73%). AKI in the first week after transplantation was related to supra-therapeutic tacrolimus concentrations (OR 1.55; 95% CI 1.06–2.27), ≥3 other nephrotoxic drugs (OR 1.96; 95% CI 1.02–3.77), infection (OR 2.48; 95% CI 1.31–4.70), and cystic fibrosis (OR 2.17; 95% CI 1.16–4.06). Recovery rate of AKI was lower than expected (19%), and the cumulative incidence of severe CKD at 1 year was 15%.

**Conclusions:**

After lung transplantation, AKI is common and often evolves into severe CKD, which is a known cause of morbidity and mortality. Supra-therapeutic whole-blood tacrolimus trough concentrations are related to the early onset of AKI. Conscientious targeting tacrolimus blood concentrations might be vital in the early phase after lung transplantation.

**Table:** 

What is known about this subject?
• Lung transplant recipients often develop acute kidney injury evolving into chronic kidney disease increasing both morbidity and mortality.
• To date, the pathophysiology of kidney injury after lung transplantation has not been fully elucidated.
• The immunosuppressant tacrolimus is difficult to dose, especially in the unstable clinical setting, and is nephrotoxic.
What this study adds:
• For the first time, supra-therapeutic whole-blood tacrolimus trough concentrations are related to the emergence of acute kidney injury in the first days after lung transplantation.
• Supra-therapeutic whole-blood tacrolimus trough concentrations often occur early after lung transplantation.
• AKI after lung transplantation shows low recovery rates.

**Electronic supplementary material:**

The online version of this article (doi:10.1007/s00228-017-2204-8) contains supplementary material, which is available to authorized users.

## Introduction

Each year, approximately 4000 lung transplantations are performed worldwide (ISHLT.org). Many of these lung transplantation patients develop acute kidney injury (AKI) [1–3]. Prevention of AKI in lung transplant recipients is vital because it is associated with the development of chronic kidney disease (CKD) increasing morbidity and mortality [2]. AKI in the first days after transplantation may be due to shock, systemic inflammation, and/or nephrotoxic drugs [4–6]. One such nephrotoxic drug is tacrolimus, a very effective immunosuppressant belonging to the calcineurin inhibitor class and ubiquitously used in lung transplant patients [7–11].

Tacrolimus nephrotoxicity in the early phase after transplantation is associated with several pharmacokinetic factors, which influence tacrolimus blood concentrations profoundly. For instance, the bioavailability of tacrolimus is highly variable due to gut dysmotility, changes in metabolism, and altered clearance due to liver injury [12]. Furthermore, tacrolimus metabolism may change with drug-drug interactions by inhibiting or competing with the transporter P-glycoprotein (Pgp) and the enzymes cytochrome P450 (CYP) 3A4/5 [13, 14]. These variations in pharmacokinetics in the early phase may result in high fluctuations in the whole-blood tacrolimus concentrations, increasing tacrolimus toxicity and decreasing tacrolimus efficacy [15, 16].

We hypothesized that tacrolimus nephrotoxicity might have a crucial role in the development of AKI after lung transplantation. Therefore, the purpose of this retrospective study was to investigate the development and recovery of kidney injury after lung transplantation and relate AKI to whole-blood tacrolimus trough concentrations.

## Patients and methods

All lung transplantation patients hospitalized at the University Medical Center Utrecht (UMCU) from July 2001 to February 2011 were retrospectively studied. Tacrolimus whole-blood trough concentrations were analyzed during the first year post transplantation. The immunosuppressive regimen consisted further of basiliximab, corticosteroids, and mycophenolate mofetil. Cofactors influencing tacrolimus blood concentrations and renal function were recorded as well. AKI was defined by the “Kidney Disease: Improving Global Outcomes” (KDIGO) Clinical Practice Guideline criteria and CKD by the “CKD Epidemiology Collaboration equation” (CKD-EPI). Both were solely based on measurement of creatinine. For more detailed information on the variables, covariates, and the used statistical analyses, see the supplemental text “Patients and Methods.”

## Results

### Patients’ characteristics

**Fig. 1:**
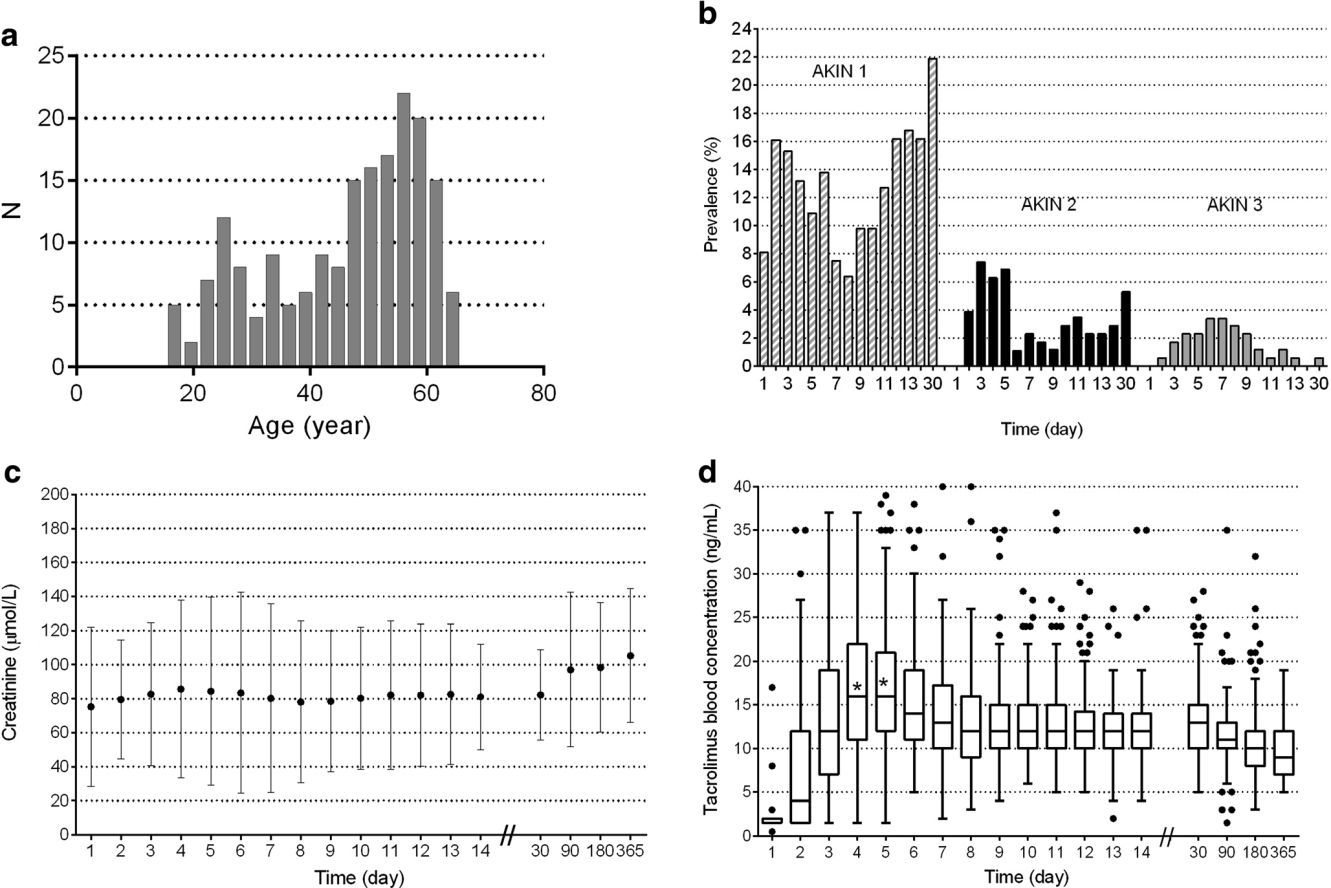
**a** Histogram of age in years. **b** Prevalence of AKI per day between day 1 and month 1 by stage in 3 stages. **c** Serum creatinine over time as mean and SD. **d** Whole-blood tacrolimus trough concentrations over time (*box*: 25th, median, and 75th percentiles); the *asterisks* in the *boxplots* show that the medians on day 4 and 5 were significantly different from 15 ng/mL on day 4 and 5

A total of 186 patients were included. Twenty-nine patients died in the first year, 11 of whom within 14 days. Patients died of bleeding (9), heart failure (1), primary graft failure (1), acute rejection (1), infection (5), hemorrhagic cerebrovascular accident (3), chronic respiratory failure (4), carcinoma (1), or an unknown cause (4). Two peaks in the age distribution were observed (Fig. 1a). The category 18–40 years contained significantly more cystic fibrosis (CF) patients than the category >40 years (*χ* ^2^ 88.09, 2 df, *p* < 0.001). Table 1 shows the patients’ characteristics. The main differences between the two groups, “AKI” or “no AKI,” involved the frequency of CF patients, the frequency of perioperative extracorporeal membrane oxygenation (ECMO), and the frequency of occurrence of infection. Further, systemic inflammatory response syndrome (SIRS) was most frequently observed on day 2 (89%, 159 out of 186) and shock on day 1 (48%, 88 out of 186). On day 6, these percentages were decreased to 34% (61 out of 186) and 5% (8 out of 186), respectively.

**Table 1:** Patients’ characteristics

Variables	All patients *N* = 186 (100%)	Follow up ≥ day 14 and no AKI day 2–14 *N* = 87 (47%)	Follow up ≥ day 14 and AKI day 2–14 *N* = 85 (46%)	*P* value^a^
	*N* (%)			
Male	91 (49%)	36 (41%)	47 (55%)	0.07
Death day 1–14	11 (6%)	0 (0%)	0 (0%)	–^b^
Death day 1–1 year	29 (16%)	1 (1%)	17 (20%)	< 0.001
Reason transplantation	*–*	*–*	*–*	0.03
CF	57 (31%)	17 (20%)	33 (39%)	0.005
COPD, emphysema, alpha-1-antitrypsin deficiency	80 (43%)	48 (55%)	28 (33%)	0.003
Sarcoidosis/ILD/UIP	14 (8%)	7 (8%)	6 (7%)	0.81
Others: PAH, IPF, bronchiectasis, allergic alveolitis, LCH, LAM	35 (19%)	15 (17%)	18 (21%)	0.51
Double transplantation	148 (80%)	64 (74%)	73 (86%)	0.045
Diabetes mellitus	40 (22%)	13 (15%)	21 (25%)	0.11
Preoperative ECMO	1 (0.5%)	1 (1%)	0 (0%)	1.00
Perioperative ECMO	118 (63%)	45 (52%)	62 (73%)	0.004
ICU admission before	19 (10%)	7 (8%)	11 (13%)	0.29
Complications	91 (49%)	35 (40%)	45 (53%)	0.10
Reoperation due to bleeding	43 (23%)	13 (15%)	20 (24%)	0.15
Infection	48 (26%)	15 (17%)	33 (39%)	0.002
Rejection	22 (12%)	7 (8%)	14 (17%)	0.09
Other	27 (15%)	9 (10%)	17 (20%)	0.08
At least once during day 1–6				
Liver injury	53 (29%)	17 (20%)	31 (37%)	0.013
Anemia	182 (98%)	85 (98%)	85 (100%)	0.50
Low protein concentration	129 (69%)	58 (67%)	65 (77%)	0.15
Supra-therapeutic whole-blood tacrolimus trough concentration	135 (73%)	61 (70%)	71 (84%)	0.04
SIRS	172 (93%)	81 (93%)	81 (95%)	0.7
Shock	115 (62%)	47 (54%)	61 (72%)	0.016
At least one drug increasing tacrolimus concentration	181 (97%)	85 (98%)	85 (100%)	0.50
At least one drug decreasing tacrolimus concentration	157 (84%)	71 (82%)	78 (92%)	0.05
Nephrotoxic drugs other than tacrolimus	178 (96%)	85 (98%)	83 (98%)	1.0
		Mean (SD)		*P* value ^a^
Age (year)	46 (13.3)	47 (12.8)	45 (13.9)	0.2
BMI	22 (3.7)	23 (3.6)	22 (3.8)	0.8

^a^Chi-square test, Fisher’s exact test, or *t* test where appropriate

^b^No statistics are computed because no death occurred

Mean baseline creatinine was 71 μmol/L (SD 35 μmol/L). Within the first 14 days after transplantation, 85 patients (46%) developed AKI (all three AKI stages pooled together) (Fig. S2). Forty-two percent of the patients (78 out of 186) presented at least one episode of AKI between day 1 and day 6. The frequency of AKI was highest on day 3 (24%, *N* = 43 out of 175). The most serious AKI (“AKI stage 3”) was especially frequent within the first 2 weeks after transplantation and occurred most often on days 6 and 7 (3.5%; 6 out of 173) (Fig. 1b). Figure 1c shows the early peak in serum creatinine with a partial decrease up to 1 month and hereafter a slow increase in serum creatinine over time. Renal replacement therapy was needed in 9% of patients on the intensive care (17 out of 186). Almost all patients received nephrotoxic drugs other than tacrolimus within the first week after transplantation (96% of patients) (Table 1). On day 2, most patients received nephrotoxic drugs other than tacrolimus (73%; 130 out of 179). The differences in patient numbers arise from transfer to another hospital, death, and the discontinuation of tacrolimus (Fig. S2).

### Variables influencing whole-blood tacrolimus concentrations

As can be seen in Fig. 1, the median whole-blood tacrolimus trough concentration first increases and then levels off. Between day 1 and day 6, 73% of patients showed supra-therapeutic concentrations (135 out of 186). Supra-therapeutic concentrations were observed most often on days 4 and 5 (50%; 87 out of 174, and 54%; 93 out of 174 of patients). At 6 months, 10% of patients (16 out of 107) showed elevated tacrolimus concentrations. Whole-blood tacrolimus trough concentrations differed significantly over time (day (estimate 1.24, 95% CI 1.02 to 1.46) and day squared (estimate −0.13, 95% CI −0.15 to −0.10)) (Table 2). Cystic fibrosis was significantly related to supra-therapeutic concentrations (estimate −0.16, 95% CI −0.28 to −0.03).

**Table 2:** Linear mixed model to test the variables influencing whole-blood tacrolimus trough concentrations

Fixed effect	Estimate^ab^	95% CI	
• CF	−0.16	−0.28	−0.03
• Liver injury	0.04	−0.08	0.16
• Drugs increasing tacrolimus	−0.02	−0.09	0.05
• ≥2 drugs increasing tacrolimus	−0.13	−0.25	0.00
• 1 or 2 drugs decreasing tacrolimus	−0.06	−0.13	0.01
• Day	1.24	1.02	1.46
• Day squared^c^	−0.13	−0.15	−0.10

^a^Estimate = regression coefficient in a linear mixed model, with log (tacrolimus concentration) as outcome variable

^b^Estimate of intercept = −0.19

^c^A quadratic term is included in the model because there was no linear relationship between outcome variable and factors included in the model

Patients often received at least one drug that could increase tacrolimus concentrations (e.g., 81% of patients on day 1 (149 out of 184) and 36% on day 5 (63 out of 173) and within 2 weeks 97%) (Table 1). At the same time, they also frequently received drugs that could decrease whole-blood tacrolimus concentrations (e.g., 63% on day 2 (112 out of 179) and 37% on day 5 (64 out of 173) and within 2 weeks 84%) (Table 1). Receiving two or more drugs, which potentially increase tacrolimus concentrations, was a significant predictor of a change in concentrations (estimate −0.13, 95% CI −0.25 to −0.00) (Table 2). The groups were too small to differentiate the distinctive drugs.

### Variables influencing AKI

The variables “supra-therapeutic whole-blood tacrolimus trough concentration” (OR 1.55, 95% CI 1.06–2.27), “infection” (OR 2.48, 95% CI 1.31–4.70), “CF” (OR 2.17, 95% CI 1.16-4.06) and " ≥3 nephrotoxic drugs other than tacrolimus" (OR 1.96, 95% CI 1.02–3.77) were all significantly associated with AKI in GEE analyses when day 2 to day 6 were concerned (Table 3). When day 2 to day 14 were incorporated, " supra-therapeutic tacrolimus concentration" (OR 1.52, 95% CI 1.04–2.24), "CF" (OR 2.23, 95% CI 1.26–4.33), and "infection" (OR 2.31, 95% CI 1.23–4.34) were significantly associated. These analyses were based on the highest level of tacrolimus obtained, regardless of the duration of the supra-therapeutic concentration.

**Table 3:** General estimating equations (GEE) analyses to test the variables influencing AKI

	Day 2–6^ac^			Day 2–14^bd^		
	OR	95% CI		OR	95% CI	
Supra-therapeutic whole-blood tacrolimus trough concentration	1.55	1.06	2.27	1.52	1.04	2.24
SIRS^e^	0.92	0.65	1.28	NA		
Shock^e^	1.56	0.82	2.95	NA		
CF	2.17	1.16	4.06	2.33	1.26	4.33
Nephrotoxic drugs other than tacrolimus^e^				NA		
1 nephrotoxic drug	2.04	0.94	4.41			
2 nephrotoxic drugs	1.40	0.73	2.69			
≥3 nephrotoxic drugs	1.96	1.02	3.77			
Double transplantation	2.07	0.77	5.54	2.15	0.81	5.69
Perioperative ECMO	1.11	0.58	2.10	1.09	0.57	2.06
Infection	2.48	1.31	4.70	2.31	1.23	4.34

^a^d2–d6: data concerning day 2 up to day 6

^b^d2–d14: data concerning day 2 up to day 14

^c^d2–d6: estimate of the intercept −2.96

^d^d2–d14: estimate of the intercept −2.82

^e^Data not available between day 7 and day 14

### Relationship between AKI, recovery, and CKD

AKI was recovered in 19% of patients (16 out of 85) at 1 month (Fig. S2). At 1 year after lung transplantation, the frequency of patients with severe CKD was 15% (23 out of 149 patients still at risk). The frequency of severe CKD for patients with “no-AKI between day 1 and day 14” was 16 out of 84 (19%), for patients with “AKI between day 1 and day 14 with recovery at 1 month” was 2 out of 14 (14%), and for patients with “AKI between day 1 and day 14 without recovery at 1 month” was 5 out of 48 patients (10%). In both groups of patients with AKI, between 14 and 25% of patients were either lost to follow-up, discontinued tacrolimus, and changed to sirolimus or had missing creatinine levels (AKI and recovery 25% (4 out of 16), AKI without recovery 14% (9 out of 64)). The mortality rate in the group of no-AKI was 1% (1 out of 87), in the group of AKI with recovery was 6% (1 out of 16), and in the group of AKI without recovery was 20% (13 out of 64). Significant differences in cumulative incidence of death were observed between the three categories of patients (*p* < 0.001). Significant differences in cumulative incidence of the combined outcome “death and severe CKD” were also observed between the three categories of patients (*p* = 0.002). The cumulative incidence of death significantly differed between groups 1 and 2 on the one hand and group 3 on the other (*p* < 0.001). The cumulative incidence of the combined outcome death and severe CKD also differed between groups 1 and 2 on the one hand and group 3 on the other (*p* = 0.001). The mortality rate and severe CKD incidence rate did not differ significantly between CF and non-CF patients (*p* = 0.77 and 0.17, respectively).

## Discussion

We draw three main conclusions from the data with respect to AKI after lung transplantation: (1) it frequently occurs in the first 14 days, (2) it shows low recovery rates and often evolves to severe chronic kidney disease, and (3) it is related to increased whole-blood tacrolimus trough concentrations.

We found high incidence rates of AKI similar to other studies on renal function in lung-transplanted patients treated with tacrolimus [2, 17]. The high occurrence rate of AKI in lung transplants might be due to a high occurrence rate of clinical instability. We related AKI to the occurrence of infection, and almost all patients exhibited SIRS and shock [2]. CF patients are especially at risk for AKI, because of a high rate of diabetes and exposure to antimicrobials, and they exhibit a high risk for postoperative complications due to a high rate of infections, hemorrhage, and perioperative ECMO use [18, 19]. We found that CF was related to supra-therapeutic tacrolimus concentrations as additional risk factor for AKI.

Apart from the high incidence rates of AKI, we observed a low rate of convalescence from AKI. Such low improvement rates of AKI have been reported previously. Wehbe et al. reported that recovery to the pretransplant renal function occurred in 34% of lung transplant patients with AKI [2]. This is in contrast to patients with septic shock, in which recovery of renal function often occurs (73% of patients) and is, in a large part, complete (60% of patients) [20]. We further observed that the cumulative incidence of death and severe CKD was significantly higher in the group of patients with AKI that had not recovered at 1 month. This is in accordance with Wehbe et al. [2]. Additionally, we observed, when no AKI occurred, that almost a fifth of patients developed severe CKD after a year. Moreover, slow deterioration of renal function may very well be related to the continuous administration of tacrolimus because it is one of the main constant nephrotoxic factors in lung transplant patients. This is in accordance with previous findings. A gradual increase of tacrolimus toxicity during the first year after transplantation has been shown in renal biopsies [21]. Especially, CYP3A5*3 carriers are associated with increased risk of kidney injury compared to CYP3A5*1 carriers. It is thought that CYP3A5*1 carriers are protected from nephrotoxicity due to a decreased exposure to tacrolimus [22–24]. Expression of CYP3A5 presumably has also a role in nephrotoxicity by directly affecting the tubular cells; reduced presence of CYP3A5 within the tubular cells increases nephrotoxicity possibly due to a diminished metabolization of tacrolimus [25].

A high whole-blood tacrolimus trough level was a risk factor for the development of AKI. Tacrolimus was above the therapeutic range in more than half of the patients in the first 6 days, which emphasizes the challenges of tacrolimus dosing in the early phase after transplantation. In particular, patients with SIRS or septic shock (the majority of patients) may have organ failure, which changes the pharmacokinetics. This, in turn, may lead to these supra-therapeutic whole-blood tacrolimus levels. Also, after recovery from clinical instability, tacrolimus dosage remains challenging. After 6 months, almost one out of ten patients still exhibited supra-therapeutic whole-blood tacrolimus trough levels.

Interestingly, the median whole-blood tacrolimus trough concentrations were not that far above the therapeutic range in the first week after transplantation. This may indicate that the whole-blood tacrolimus trough concentrations were not the only contributing factor to tacrolimus toxicity. We hypothesize that the unbound tacrolimus plasma concentrations are potentially more responsible for the nephrotoxicity than the whole-blood tacrolimus concentrations. Only the unbound plasma concentration of a drug is biologically active and potentially toxic. Tacrolimus is highly bound to erythrocytes, albumin, α1-acid glycoprotein (AGP), and high-density lipoprotein (HDL) in stable clinical conditions, though the capacity of tacrolimus to bind may widely fluctuate in times of systemic inflammation and shock [26]. Erythrocytes and proteins are known to extensively vary during clinical instability [27, 28]. Unfortunately, the unbound tacrolimus plasma concentrations cannot be measured by routine analyses. Consequently, knowledge of unbound tacrolimus plasma concentrations is scarce as well as its relation to nephrotoxicity. Our data showed large decreases in the number of erythrocytes and protein levels in the majority of patients in the first week after transplantation (See Table S3). These decreases may have influenced unbound plasma concentrations without having an effect on whole-blood concentrations. Therefore, the unbound tacrolimus plasma concentrations may be a more sensitive biomarker of nephrotoxicity.

### Remarks regarding this study

There are some limitations to this study due to the retrospective design. Several explanatory variables influencing the pharmacokinetics of tacrolimus could not be investigated because of the number of missing values. For instance, the effect of variations in variables like acidosis, changes in fluid balance, gut motility, or variations in concentrations and activity of CYP 3A4/5 and P-glycoprotein could not be examined. They all may have an effect on whole-blood tacrolimus concentrations and should be considered as residual confounders.

The whole-blood tacrolimus trough concentrations were measured at approximately 12 h after administration. Tacrolimus elimination half-life time and time to trough level may substantially variate in solid organ recipients [29]. Therefore, the “apparent” trough levels monitored and used for dose adjustments according to the current practice may not always reflect optimal trough levels [30].

Cystic fibrosis patients showed unexpectedly higher whole-blood tacrolimus concentrations. Cystic fibrosis patients generally have a decreased bioavailability and higher phase II metabolism resulting in a lower area under the concentration-time curve [31, 32]. Whether these CF patients received higher doses or had a decreased metabolism due to variations in CYP3A4/5 or P-glycoprotein gene expression and drug-drug interactions could not be determined.

Different definitions of renal function complicate the comparison of the results with other studies [1, 17, 33, 34]. To allow for a better comparison, we analyzed our data with the criteria used by Wehbe et al., i.e., the KDIGO criteria were used without the urine output [2]. Plasma creatinine levels in lung transplantation patients after surgery may overestimate renal function due to pronounced muscle loss and depressed production of creatinine, which may result in lower creatinine plasma concentrations and an underestimation of the percentage of kidney injury [35, 36].

Investigations like ultrasound, biopsy, and urine analyses are not performed at a regular basis in clinically unstable lung transplant patients. Therefore, other causes of kidney injury are not excluded in this cohort and may have had an effect on kidney function. All AKI was attributed to tacrolimus, and this might lead to an overestimation of the nephrotoxicity caused by tacrolimus. Important alternatives are the predisposing factors for AKI, which are shown in Table 1.

## Conclusions

AKI is common after lung transplantation and is associated with both morbidity and mortality. We related supra-therapeutic whole-blood tacrolimus concentrations, next to three or more other nephrotoxic drugs, CF, and infection to AKI in the first week after transplantation. There was a high occurrence of hemodynamic instability perioperatively. Whereas hemodynamic instability is a known cause of AKI, recovery to pretransplant renal function is expected. We observed an ongoing deterioration of renal function even when patients were considered stable. We related supra-therapeutic whole-blood tacrolimus concentrations early after transplantation to the emergence of AKI. This study underlines the significance of unraveling tacrolimus pharmacokinetics early after transplantation in order to decrease AKI in this vulnerable group of patients.

### Authors’ contributions:

All authors made substantial intellectual contributions to conception and design (M.A.S., C.C.H., J.M.), acquisition of data and data analysis (M.A.S., C.C.H.), and writing or interpretation of the data (M.A.S., C.C.H., E.A.G., M.C.V., J.K., D.W.L., and J.M.). All authors contributed to drafting the article or revising it critically for important intellectual content and gave final approval of the version to be published and approved of the order in which their names will be listed in the manuscript. M.A.S. and C.C.H. had full access to all data in the study and take responsibility for the integrity of the data and the accuracy of the data analysis.

## Electronic supplementary material

ESM 1 (DOCX 267 kb)

## Abbreviations

ACCP: American College of Chest Physicians
AGP: α1-Acid glycoprotein
AKI: Acute kidney injury
AKIN: Acute Kidney Injury Network
ALAT: Alanine aminotransferase
Alb: Albumin
BMI: Body mass index
CF: Cystic fibrosis
CI: Confidence interval
CKD: Chronic kidney disease
CKD-EPI: CKD Epidemiology Collaboration
COPD: Chronic obstructive pulmonary disease
CYP: Cytochrome P450
ECMO: Extracorporeal membrane oxygenation
GEE: Generalized estimating equation
GFR: Glomerular filtration rate
Hb: Hemoglobin
HDL: High-density lipoprotein
Ht: Hematocrit
IPF: Idiopathic pulmonary fibrosis
LAM: Lymphangioleiomyomatosis
LCH: Langerhans cell histiocytosis
OR: Odds ratio
PAH: Pulmonary arterial hypertension
Pgp: P-glycoprotein
SCCM: Society of Critical Care Medicine
SD: Standard deviation from the mean
SIRS: Systemic inflammatory response syndrome
TGF-β1: Transforming growth factor β1
