# Supplementary material for: High tacrolimus blood concentrations early after lung transplantation and the risk of kidney injury
Source: Eur J Clin Pharmacol. 2017 Jan 28;73(5):573–80. doi: 10.1007/s00228-017-2204-8 (PMC5384949; doi:10.1007/s00228-017-2204-8)
Supplement: Supplementary file 1 — (DOCX 267 kb) [file 228_2017_2204_MOESM1_ESM.docx]

**Supplementary material**

**High tacrolimus blood concentrations early after lung transplantation and the risk of kidney injury**

**M. A. Sikma^1,2^, C. C. Hunault^2^, E. A. van de Graaf^3^, M. C. Verhaar^4^, J. Kesecioglu^1^, D. W. de Lange^1,2^, J. Meulenbelt^1,2,5^†**

**1. Department of Intensive Care Medicine, University Medical Center Utrecht, Utrecht, The Netherlands**

**2. Dutch Poisons Information Center, University Medical Center Utrecht, Utrecht, The Netherlands**

**3. Department of Lung Transplantation, University Medical Center Utrecht, Utrecht, The Netherlands**

**4. Department of Nephrology and Hypertension, University Medical Center Utrecht, Utrecht, the Netherlands**

**5. Institute for Risk Assessment Sciences, University Medical Center Utrecht, The Netherlands**

**† Prof. J Meulenbelt, MD, PhD died in December 2015**

This webappendix formed part of the original submission and has been peer reviewed.

We post it as supplied by all authors.

Index

[PATIENTS AND METHODS 3](#_Toc472322848)

[Patients and therapy 3](#_Toc472322849)

[Definitions of acute kidney injury (AKI) and chronic kidney disease (CKD) (See Table S1) 4](#_Toc472322850)

[Definitions of the covariates (See Table S1) 4](#_Toc472322851)

[Statistical analyses (See Figure S1 and Table S2) 4](#_Toc472322852)

[Variables influencing the tacrolimus whole-blood trough concentrations 5](#_Toc472322853)

[Variables influencing AKI 5](#_Toc472322854)

[Relationship between AKI, recovery and CKD 5](#_Toc472322855)

[Figure S1 6](#_Toc472322856)

[Table S1 8](#_Toc472322857)

[Table S2 9](#_Toc472322858)

[Table S3 11](#_Toc472322859)

# PATIENTS AND METHODS

The study was conducted in compliance with the 2008 Declaration of Helsinki and Good Clinical Practice guidelines and with local and national regulatory requirements and laws. The accredited review board for human studies of the UMCU approved the study (IRB protocol number 11-357/G-C).

## Patients and therapy

All lung transplantation patients hospitalized at the UMCU from July 2001 to February 2011 were retrospectively studied. Patients were analyzed from the transplantation date until one year after transplantation. Tacrolimus was orally dosed twice daily from the first postoperative day onwards with a starting dose of 0.1 mg/kg. Adjustments on basis of interactions with other drugs, gut dysmotility and liver injury were left to the discretion of the attending physician. Additional dosing was based on daily whole-blood tacrolimus trough concentrations at 6 am (C12h). Dose adjustments were often made on a daily basis and steady state was not necessarily reached at the time of dose adjustments. A whole-blood tacrolimus trough concentration between 9 and 15 ng/mL was considered therapeutic [1]. Although tacrolimus trough concentrations were measured daily we included only the tacrolimus blood concentrations that were taken at day 1 to 14 and at 1, 3, 6 and 12 months after transplantation. The tacrolimus blood concentrations were analyzed using a micro-particle enzyme immunoassay (Abbott IMx™). The immunosuppressive regimen consisted further of basiliximab induction therapy on day 1 and 4 post-operative [20 mg intravenously], corticosteroids [prednisolon 25 mg per day qid intravenously and tapered off to 25 mg od orally after four days] and mycophenolate mofetil [starting dose 1500 mg orally bid, tapered off to 1000 mg bid]. The administration of the following potentially nephrotoxic drugs other than tacrolimus was recorded as a categorical variable with more categories (0, 1, 2 or ≥3): (val)acyclovir, (val)ganciclovir, tobramycin, gentamicin, furosemide, vancomycin and amphotericin B. Other factors potentially related to kidney injury were recorded as well, such as septic shock, systemic inflammatory response syndrome (SIRS), diagnosis of sarcoidosis, cystic fibrosis (CF), chronic obstructive pulmonary disease (COPD), or alpha-1-antitrypsin deficiency, diabetes, body mass index (BMI), pre-transplant renal function, perioperative extracorporeal membrane oxygenation (ECMO), double lung transplantation, and also postoperative profound bleeding for which reoperation was needed, infection and acute rejection [2].

## Definitions of acute kidney injury (AKI) and chronic kidney disease (CKD) (See Table S1)

AKI was classified according to the “Kidney Disease: Improving Global Outcomes” (KDIGO) Clinical Practice Guideline, which distinguishes 3 stages [3]. These stages were solely based on serum creatinine concentration because urine data were unavailable. Classification was determined by the most severe stage of AKI. Indications for renal replacement therapy were stage 3 combined with hyperkalemia, untreatable hypervolemia, uncorrectable metabolic acidosis and severe azotemia. A serum creatinine lower or equal to the baseline creatinine + 5% indicated renal recovery after an AKI event. CKD was determined according to the estimated GFR using the “CKD Epidemiology Collaboration equation (CKD EPI)” [4]. Albuminuria was not known. Therefore, only serum creatinine was used for categorizing CKD. Severe CKD was defined as having a stage 4 or 5 and was evaluated after 3 and 6 months and 1 year.

## Definitions of the covariates (See Table S1)

SIRS has been defined according to the definition of the American College of Chest Physicians (ACCP) and the Society of Critical Care Medicine Consensus Conference (SCCM) [5]. Patients considered to have septic shock had SIRS in combination with infusion of at least one inotrope/vasopressive agent. Liver injury was defined as a bilirubin >34 μmol/L or an alanine aminotransferase (ALAT) >90 U**/**L for men and >70 U/L for women [6]. Drugs influencing tacrolimus pharmacokinetics were also recorded: drugs that potentially increase tacrolimus blood concentrations by inhibition or substrate competition of the enzymes CYP3A4/5 and transporter Pgp and drugs that potentially decrease tacrolimus blood concentrations by induction of the enzymes CYP3A4/5 or transporter Pgp [7].

## Statistical analyses (See Figure S1 and Table S2)

Statistical analyses were executed using SPSS version 15.0 for Windows (SPSS® Inc., Chicago, USA) and SAS version 9.2 for Windows (SAS® Institute Inc., Cary, NC, USA). Variables are presented as mean (standard deviation (SD)), median (25th and 75th percentiles), Odds ratio (OR) or estimate with 95% confidence interval (CI) or number (proportion) where appropriate. Chi-square tests or Fisher’s exact tests were used to test differences between groups of patients for categorical data. T-tests or Wilcoxon Rank Tests were used for continuous data.

## Variables influencing the tacrolimus whole-blood trough concentrations

Mixed model analyses were applied to investigate the variables influencing the whole-blood tacrolimus trough concentrations. Analyses were performed using the “PROC MIXED option” in SAS (See Figure S1 and Table S2). The patient’s identification number was the subject variable. Different variables (Cystic Fibrosis, liver dysfunction, drugs possibly increasing tacrolimus blood concentrations and drugs possibly decreasing tacrolimus blood concentrations) were tested independently and included as fixed factors. Time was included as both a within-subject and a between-subject variable.

## Variables influencing AKI

In longitudinal data with a dichotomous outcome, outcomes within one patient are correlated. To account for this within-patient effect, we applied Generalized Estimation Equation (GEE) analyses [8]. The “PROC GENMOD” in SAS was used to study whether a high whole-blood tacrolimus trough concentration, SIRS, septic shock and the administration of a nephrotoxic drug, not being tacrolimus, were predictive of AKI up to 6 and 14 days after transplantation.

## Relationship between AKI, recovery and CKD

Kaplan-Meier analyses were performed to study the probability of surviving without CKD up to one year. We distinguished three groups of patients: group 1 included patients without AKI between day 1 and day 14; group 2 included patients having AKI between day 1 and day 14 with recovery at 1 month; and group 3 included patients with AKI between day 1 and day 14 without recovery at 1 month. Differences between the three groups of patients were tested using the log rank test. Differences between CF and non-CF patients were tested using the same method.

**References**

1. Monchaud C, Marquet P. Pharmacokinetic optimization of immunosuppressive therapy in thoracic transplantation: part I. Clinical Pharmacokinetics. Springer International Publishing; 2009;48(7):419–62.

2. Grimm JC, Lui C, Kilic A, Valero V, Sciortino CM, Whitman GJR, et al. A risk score to predict acute renal failure in adult patients after lung transplantation. Ann Thorac Surg. 2015 Jan;99(1):251–7.

3. Kellum JA, Lameire N, KDIGO AKI Guideline Work Group. Diagnosis, evaluation, and management of acute kidney injury: a KDIGO summary (Part 1). Crit Care. BioMed Central; 2013;17(1):204.

4. Levey AS, Stevens LA, Schmid CH, Zhang YL, Castro AF, Feldman HI, et al. A new equation to estimate glomerular filtration rate. Ann Intern Med. NIH Public Access; 2009 May 5;150(9):604–12.

5. Bone RC, Balk RA, Cerra FB, Dellinger RP, Fein AM, Knaus WA, et al. Definitions for sepsis and organ failure and guidelines for the use of innovative therapies in sepsis. The ACCP/SCCM Consensus Conference Committee. American College of Chest Physicians/Society of Critical Care Medicine. 1992. pp. 1644–55.

6. Baran DA, Galin ID, Zucker MJ, Alvi S, Arroyo LH, Lubitz S, et al. Can initial tacrolimus trough levels be predicted from clinical variables? Transplantation Proceedings. 2004 Nov;36(9):2816–8.

7. Sikma MA, van Maarseveen EM, van de Graaf EA, Kirkels JH, Verhaar MC, Donker DW, et al. Pharmacokinetics and Toxicity of Tacrolimus Early After Heart and Lung Transplantation. Am J Transplant. 2015 Jun 4;15(9):2301–13.

8. Zeger SL, Liang KY. Longitudinal data analysis for discrete and continuous outcomes. Biometrics. 1986 Mar;42(1):121–30.

# Figure S1

Performed statistical analyses: 1- Linear mixed model, 2- GEE analyses and 3- Kaplan-Meier analyses

**
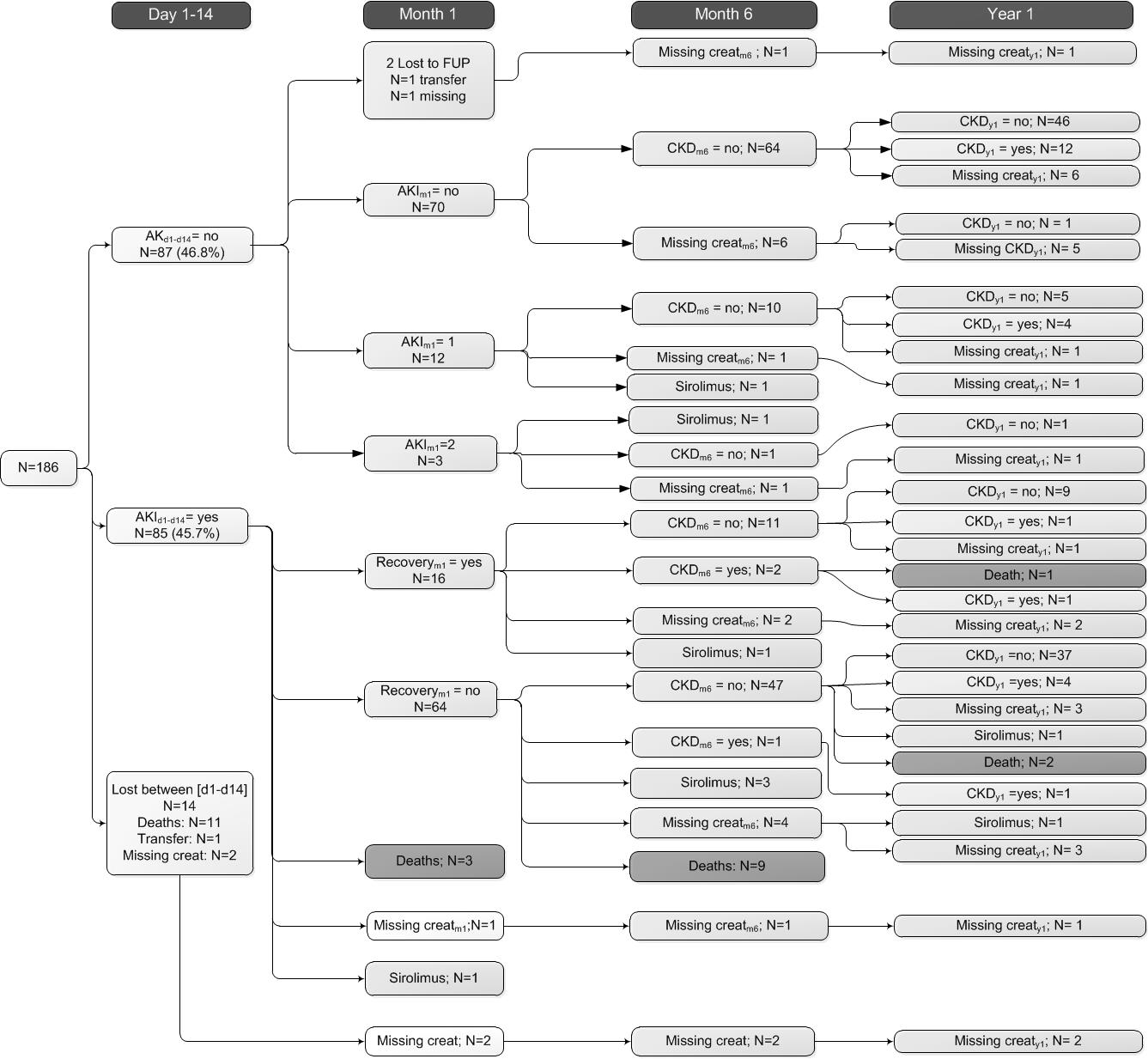
**Fig. S2

Flow-chart: Frequency of AKI and severe chronic kidney disease between day 1 and year 1

| Table S1 | |
| --- | --- |
| Definitions of the covariates | |
| **Covariate** | **Definition** |
| AKI  CKD | “No AKI” ; No increase in serum creatinine from baseline or serum creatinine <354 μmol/L. Stage 1; Increase in serum creatinine ≥26 μmol/L or 150-200% from baseline. Stage 2; Increase in serum creatinine >200% and ≤300% from baseline. Stage 3; Increase in serum creatinine >300% or ≥354 μmol/L with an acute increase of minimally 44 μmol/L or initiation of renal replacement therapy  GFR categories; G1=normal GFR: ≥90 mL/min /1.73m^2^, G2=mildly decreased: 60–89 mL/min /1.73m^2^, G3a/b=mildly to severely decreased: 30-59 mL/min /1.73m^2^, G4= severely decreased: 15-29 mL/min /1.73m^2^, G5= Kidney failure: <15 mL/min/1.73m^2^. GFR = 141 x [min(Scr/κ),1)α x max(Scr/κ),1)-1.209] x  Age-0.993 x 1.018 [if female] x [1.157 if Black]  α is 0.329 for females and 0.411 for males; min indicates minimum of Scr/κ or 1, and max indicates maximum of Scr/κ or 1 |
| SIRS | Presenting 2 or more of the following criteria: body temperature <36 °C or >38 °C, heart rate >90/min, respiratory rate >20/min, PaCO2 <32 mmHg, mechanical ventilation and leucocyte count <4 X10^9^/L or >12 X10^9^/L |
| Septic shock | SIRS plus at least one inotrope/ vasopressant (norepinephrine, dopamine, dobutamine and milrinone) |
| Liver injury | Bilirubin >34 μmol/L or an ALAT >90 U/L for men and >70 U/L for women |
| Nephrotoxic drugs other than tacrolimus | Tobramycin, vancomycin, (val)aciclovir, (val)ganciclovir, furosemide, Amfo B |
| Drugs increasing tacrolimus blood concentrations by inhibition or substrate competition of the CYP3A4/5 and Pgp enzymes | Basiliximab, erythromycin, fluconazole, voriconazole, (es)omeprazole, amlodipine, nicardipine, diltiazem, haloperidol and amiodarone |
| Drugs potentially decreasing tacrolimus blood concentrations by induction of CYP3A4/5 or Pgp enzymes | Corticosteroids and rifampicin |

| Table S2 | | | |
| --- | --- | --- | --- |
| Type of analysis used for the different outcome variables with the potential confounders | | | |
| **Type of analysis** | **Outcome variable** | **Tested variable(s)** | **Potential confounder(s)** |
| Linear Mixed model | Whole-blood tacrolimus trough concentration^1^ | - CF - Liver injury   Other drugs increasing tacrolimus concentration  Other drugs decreasing tacrolimus concentration^2^ |  |
| GEE analysis^3^ | AKI between day 2-6 | - Supra-therapeutic whole-blood tacrolimus trough concentrations day 2-6 | - SIRS - Shock - CF   Nephrotoxic drugs other than tacrolimus   - Double lung transplantation - Perioperative ECMO - Infection |
|  | AKI between day 2-14 | - Supra-therapeutic whole-blood tacrolimus trough concentrations day 2-14 | - CF   Double lung transplantation  Perioperative ECMO  Infection |
| Kaplan-Meier analyses | Severe CKD up to 1 year | - CF - AKI between day 2-14 ± recovery at 1 month |  |
| ^1^The tacrolimus whole-blood concentration was log transformed for fitting purposes.  ^2^Fixed factors were: CF, liver injury, , the number of drugs possibly decreasing the tacrolimus concentration and the number of drugs possibly increasing the tacrolimus concentration, included as categorical variables. The effect of drugs on the tacrolimus concentration was tested one day after their initiation. Observations were clustered within individuals (patients’ identification number as subject variable) and time (expressed in day) was entered as both a within-subject and a between-subject variable. A quadratic term for day had to be included in the model for fitting purposes as the relationship between whole-blood tacrolimus concentration and day was non-linear.  ^3^A binary logistic model was selected, with a logit link and an exchangeable working matrix. The outcome variable was “AKI”, with two categories: “normal” corresponding to the no AKI and “abnormal” corresponding to the AKIN stages 1, 2, and 3 together. The significance of the variables was tested by Wald chi-square tests. | | | |

| Table S3 Concentrations of Hb, Ht, albumin and total protein day 1 to 6 | | | | | |
| --- | --- | --- | --- | --- | --- |
|  | | - Hb^1,2^ mmol/L | - Ht^1,2^ | - Albumin^1,3^ g/L | - Total Protein^1,3^ g/L |
| - Median | | - 6.1 | - .29 | - 23.2 | - 47 |
| - Percentile | - 25th | - 5.3 | - .25 | - 19.4 | - 41 |
|  | - 75th | - 6.8 | - .33 | - 26.5 | - 53 |
|  |  |  |  |  |  |
| ^1^Anemia was defined as Ht <0.35 or Hb <7 mmol/L and low protein as albumin <20 g/L or total protein concentration <45 g/L.  ^2^Anemia was observed in 98% of patients (182 out of 186).  ^3^A low protein level was found in 69% of patients (129 out of 186). | | | | | |
